# Supplementary material for: Exploiting somatic piRNAs in Bemisia tabaci enables novel gene silencing through RNA feeding
Source: Life Sci Alliance. 2020 Aug 6;3(10):e202000731. doi: 10.26508/lsa.202000731 (PMC7425214; doi:10.26508/lsa.202000731)
Supplement: Supplementary file 4 [file LSA-2020-00731_Supplemental_Data_1.docx]

**Supplementary Information for**

**Exploiting somatic piRNAs in the whitefly, *Bemisia tabac*i** **enables a new mode of gene silencing through synthetic RNA feeding**

Mosharrof Mondal^1^, Judith K. Brown^1^, Alex Flynt^2,*^

^1^School of Plant Sciences, University of Arizona, Tucson, Arizona, 85721, United States of America

^2^Cellular and Molecular Biology, University of Southern Mississippi, Hattiesburg, Mississippi, 39406, United States of America

*Corresponding author: Alex Flynt

Email: [alex.flynt@usm.edu](mailto:alex.flynt@usm.edu)

Supplementary Text

**Whitefly phusion constructs synthesis and cloning**

**PCR recipe for SOEing PCR**

Phire Plant Direct PCR Master Mix 25 uL

Phire dilution buffer 1.5 ul

Fragment A (25ng)

Fragment B (25ng)

ddH_2_O make the reaction to 50 uL

**Thermocycler steps**

98^o^C 5 min

}

98^o^C 7 sec

10 cycle

53 ^o^C 10 sec

72^o^C 7 sec

72^o^C 5 min

After the first 10 cycles, end primers were added and the thermocycler was ran for another 25 cycles following these steps:

98^o^C 5 min

}

98^o^C 7 sec

25 cycle

63 ^o^C (Annealing Tm) 10 sec

72^o^C 15 sec

72^o^C 5 min

Phire DNA polymerase doesn’t create any ‘A’ overhang. For T-A cloning into pGEM-T easy vector, the ‘A’ nt was added to the final fusion products using *Taq* DNA polymerase.

**Sequences of the constructs**

Capital letter sequences are piRNA trigger, sandwiched lower case sequences are from gene of interest (AQP1, AGLU1, Luciferase)

**AQP1-piRB-6**

AGCAGCTTCTTGCCTCTGATTCCACGGTTTCTTCTTAAAGGGCCCCGACGACTGCTGCGGGCCTTGATAAGGCGCGCTCCTGTTATTTGCCTCACGGAACGTCTTTTCCGCGGCCATCATTGCGTCCATTGATCGGATCAAATCTTGCCTCATTGCATCCACGGCTCGAGTATTCCTATCCGTATCCGCACGATTTAGATCAACTGCGTGTACCAAAGTCGCTAGGGCGTTCTCATTGGCCTTCACCCGGGATTCTAAGGATGATTCCTGCCCCGTATAGTGATTTACGGCCAAAATAGCGCCCCTTCCTTTGCTGGTCGCGGCTACTGCTAGCTTCGCATTtcgcacaatgccttggagccatctgtggagcaatcattctgaatgaaatcacgccaaaaacaggttacacggctgctggtaatctgggagtaacgacactgtctacaggagtttccgacctgcagggtgtggcgatagaagcactaatcacatttgtgctgcttttagttgtccagtccgtctgcgatgggaagcggaccgacatcaaaggatctatcggcgttgcgataggattcgcaattgctTCCGTCGAGTTAACTTTAGCCAAGCCCGCTAGTTTTCTCTTCGCTTGAACGTAATCCAACGGGTCCTCATTTTCTCCCTGCGTTCGCGCCGAGAATTTCGTGAGGGCATCCTCGTCGCTGTCAAAGTATTGGATCAATTTCTTCTTTACTTCCTCAAAAGTCCTGCAGTTACCGAACGCTACCTCTTCATTGTCGTAGTACTGGATGGCACGTTTCGCTAAGTGATTTCTGAGTTGGTCCCGCTTTTCTTGATCCGAACATTTCTTATAGAAATTTTCAAAATCTTTTAGAAATTCTCTAACGTCGTAGTCAGCTTCTCCTTTGAATAGTTTTCTAAACGGCGGTGCCTTAATCGTCACCGTAGGT

**AQP1-No_bias-14**

TTGCGTTCCTGCTCCCTTTGCCCTTTACCGCGCTCAATTATCTCTATTAGAACCGGAGATATTCGGTTTACAAAAATTTTTTGGGGCCCAGCCCCCCTTAATCCTTTCCCTATGGACTTCCTATATGGCCCCAGAGGTAGCCCCCGGGGGTTAGGCAAATAATCCCAAAAAATTCCCAAATTCTAACGGAAATGTGGCACTACCGCCCCTACGTCACTCTGGCTATGACGTAGTTGATtcgcacaatgccttggagccatctgtggagcaatcattctgaatgaaatcacgccaaaaacaggttacacggctgctggtaatctgggagtaacgacactgtctacaggagtttccgacctgcagggtgtggcgatagaagcactaatcacatttgtgctgcttttagttgtccagtccgtctgcgatgggaagcggaccgacatcaaaggatctatcggcgttgcgataggattcgcaattgctTTACGTGCCGTTACACCGGTTACCGACATCAGGTTCCTTCAAATCGGACACGGGCGCCCCTCCCCGAGGGGATGCCAATGGGGGGAGGTCCCAGGCCGAAGCCTGACTTTCTACTACCTCCGGAGCTGTGCCCTTCTCTGCACGTCCCAGTTGAGCACTGGTGGGCTGACCTCGGGGACAAGGTCGCCTTAACTTACCG

**AGLU1-piRB-6**

AGCAGCTTCTTGCCTCTGATTCCACGGTTTCTTCTTAAAGGGCCCCGACGACTGCTGCGGGCCTTGATAAGGCGCGCTCCTGTTATTTGCCTCACGGAACGTCTTTTCCGCGGCCATCATTGCGTCCATTGATCGGATCAAATCTTGCCTCATTGCATCCACGGCTCGAGTATTCCTATCCGTATCCGCACGATTTAGATCAACTGCGTGTACCAAAGTCGCTAGGGCGTTCTCATTGGCCTTCACCCGGGATTCTAAGGATGATTCCTGCCCCGTATAGTGATTTACGGCCAAAATAGCGCCCCTTCCTTTGCTGGTCGCGGCTACTGCTAGCTTCGCATTCtgtccatccaaccctggattgccttttggtaatctttggcgggagagcgaccgctcacgtgcgtaataaagaagaaattgaatggcatatgggctcctggtttcccctcaaactgataatagtccattgttctatcgagagttgtatatgcttctgtcattagtacttttgttttcccctctttctttgtgtagaaatcgaagacctccctgaaccttgttatcaatctatatgtattcggttggtccatcgtccgcgaccggttgtaattccagtagttcgtcgggtcgagatcgggcgagagcaattcttggtctcgccattTCCGTCGAGTTAACTTTAGCCAAGCCCGCTAGTTTTCTCTTCGCTTGAACGTAATCCAACGGGTCCTCATTTTCTCCCTGCGTTCGCGCCGAGAATTTCGTGAGGGCATCCTCGTCGCTGTCAAAGTATTGGATCAATTTCTTCTTTACTTCCTCAAAAGTCCTGCAGTTACCGAACGCTACCTCTTCATTGTCGTAGTACTGGATGGCACGTTTCGCTAAGTGATTTCTGAGTTGGTCCCGCTTTTCTTGATCCGAACATTTCTTATAGAAATTTTCAAAATCTTTTAGAAATTCTCTAACGTCGTAGTCAGCTTCTCCTTTGAATAGTTTTCTAAACGGCGGTGCCTTAATCGTCACCGTAGGT

**AGLU1-No_bias-14**

TTGCGTTCCTGCTCCCTTTGCCCTTTACCGCGCTCAATTATCTCTATTAGAACCGGAGATATTCGGTTTACAAAAATTTTTTGGGGCCCAGCCCCCCTTAATCCTTTCCCTATGGACTTCCTATATGGCCCCAGAGGTAGCCCCCGGGGGTTAGGCAAATAATCCCAAAAAATTCCCAAATTCTAACGGAAATGTGGCACTACCGCCCCTACGTCACTCTGGCTATGACGTAGTTGATctgtccatccaaccctggattgccttttggtaatctttggcgggagagcgaccgctcacgtgcgtaataaagaagaaattgaatggcatatgggctcctggtttcccctcaaactgataatagtccattgttctatcgagagttgtatatgcttctgtcattagtacttttgttttcccctctttctttgtgtagaaatcgaagacctccctgaaccttgttatcaatctatatgtattcggttggtccatcgtccgcgaccggttgtaattccagtagttcgtcgggtcgagatcgggcgagagcaattcttggtctcgccattTTACGTGCCGTTACACCGGTTACCGACATCAGGTTCCTTCAAATCGGACACGGGCGCCCCTCCCCGAGGGGATGCCAATGGGGGGAGGTCCCAGGCCGAAGCCTGACTTTCTACTACCTCCGGAGCTGTGCCCTTCTCTGCACGTCCCAGTTGAGCACTGGTGGGCTGACCTCGGGGACAAGGTCGCCTTAACTTACCG

**Luciferase-piRB-6**

AGCAGCTTCTTGCCTCTGATTCCACGGTTTCTTCTTAAAGGGCCCCGACGACTGCTGCGGGCCTTGATAAGGCGCGCTCCTGTTATTTGCCTCACGGAACGTCTTTTCCGCGGCCATCATTGCGTCCATTGATCGGATCAAATCTTGCCTCATTGCATCCACGGCTCGAGTATTCCTATCCGTATCCGCACGATTTAGATCAACTGCGTGTACCAAAGTCGCTAGGGCGTTCTCATTGGCCTTCACCCGGGATTCTAAGGATGATTCCTGCCCCGTATAGTGATTTACGGCCAAAATAGCGCCCCTTCCTTTGCTGGTCGCGGCTACTGCTAGCTTCGCATTttcgtgccagagtctttcgacagggacaaaaccattgccctgatcatgaacagctctgggtctaccggcctgcctaagggcgtggccctgcctcatcgcaccgcctgtgtgcgcttctctcacgcccgcgaccctattttcggcaaccagatcatccccgacaccgctattctgagcgtggtgccattccaccacggcttcggcatgttcaccaccctgggctacctgattTCCGTCGAGTTAACTTTAGCCAAGCCCGCTAGTTTTCTCTTCGCTTGAACGTAATCCAACGGGTCCTCATTTTCTCCCTGCGTTCGCGCCGAGAATTTCGTGAGGGCATCCTCGTCGCTGTCAAAGTATTGGATCAATTTCTTCTTTACTTCCTCAAAAGTCCTGCAGTTACCGAACGCTACCTCTTCATTGTCGTAGTACTGGATGGCACGTTTCGCTAAGTGATTTCTGAGTTGGTCCCGCTTTTCTTGATCCGAACATTTCTTATAGAAATTTTCAAAATCTTTTAGAAATTCTCTAACGTCGTAGTCAGCTTCTCCTTTGAATAGTTTTCTAAACGGCGGTGCCTTAATCGTCACCGTAGGT

**Luciferase-No_bias-14**

TTGCGTTCCTGCTCCCTTTGCCCTTTACCGCGCTCAATTATCTCTATTAGAACCGGAGATATTCGGTTTACAAAAATTTTTTGGGGCCCAGCCCCCCTTAATCCTTTCCCTATGGACTTCCTATATGGCCCCAGAGGTAGCCCCCGGGGGTTAGGCAAATAATCCCAAAAAATTCCCAAATTCTAACGGAAATGTGGCACTACCGCCCCTACGTCACTCTGGCTATGACGTAGTTGATttcgtgccagagtctttcgacagggacaaaaccattgccctgatcatgaacagctctgggtctaccggcctgcctaagggcgtggccctgcctcatcgcaccgcctgtgtgcgcttctctcacgcccgcgaccctattttcggcaaccagatcatccccgacaccgctattctgagcgtggtgccattccaccacggcttcggcatgttcaccaccctgggctacctgattTTACGTGCCGTTACACCGGTTACCGACATCAGGTTCCTTCAAATCGGACACGGGCGCCCCTCCCCGAGGGGATGCCAATGGGGGGAGGTCCCAGGCCGAAGCCTGACTTTCTACTACCTCCGGAGCTGTGCCCTTCTCTGCACGTCCCAGTTGAGCACTGGTGGGCTGACCTCGGGGACAAGGTCGCCTTAACTTACCG

**AQP1 sequence used in this study to synthesize dsRNA (from accession # KF377800.1)**

tcgcacaatgccttggagccatctgtggagcaatcattctgaatgaaatcacgccaaaaacaggttacacggctgctggtaatctgggagtaacgacactgtctacaggagtttccgacctgcagggtgtggcgatagaagcactaatcacatttgtgctgcttttagttgtccagtccgtctgcgatgggaagcggaccgacatcaaaggatctatcggcgttgcgataggattcgcaattgct

**AGLU1 sequence used in this study to synthesize dsRNA (from accession # KF377803.1)**

ctgtccatccaaccctggattgccttttggtaatctttggcgggagagcgaccgctcacgtgcgtaataaagaagaaattgaatggcatatgggctcctggtttcccctcaaactgataatagtccattgttctatcgagagttgtatatgcttctgtcattagtacttttgttttcccctctttctttgtgtagaaatcgaagacctccctgaaccttgttatcaatctatatgtattcggttggtccatcgtccgcgaccggttgtaattccagtagttcgtcgggtcgagatcgggcgagagcaattcttggtctcgccatt

**Luciferase sequence was cloned from** psiCHECK™-2 plasmid (Promega, catalog # C8021)

ttcgtgccagagtctttcgacagggacaaaaccattgccctgatcatgaacagctctgggtctaccggcctgcctaagggcgtggccctgcctcatcgcaccgcctgtgtgcgcttctctcacgcccgcgaccctattttcggcaaccagatcatccccgacaccgctattctgagcgtggtgccattccaccacggcttcggcatgttcaccaccctgggctacctgatt

**Primer sequences used in this study**

**(**Underlined regions are overlap to the genes of interest)

**piB6_A F**

AGCAGCTTCTTGCCTCTGATTCCAC

**piB6_B R**

AATGCGAAGCTAGCAGTAGCCGC

**piB6_C F**

TCCGTCGAGTTAACTTTAGCCAAGCC

**piB6_D R**

ACCTACGGTGACGATTAAGGCACC

**Eq14_A F**

TTGCGTTCCTGCTCCCTTTGCC

**Eq14_B R**

ATCAACTACGTCATAGCCAGAGTGACG

**Eq14_C F**

TTACGTGCCGTTACACCGGTTACC

**Eq14_D R**

CGGTAAGTTAAGGCGACCTTGTCCC

**AQP1-piB6 F**

GCTGGTCGCGGCTACTGCTAGCTTCGCATTTCGCACAATGCCTTGGAGCCATC

**AQP1-piB6 R**

AGCGGGCTTGGCTAAAGTTAACTCGACGGAAGCAATTGCGAATCCTATCGCAACG

**AQP1-Eq14 F**

CTACGTCACTCTGGCTATGACGTAGTTGATTCGCACAATGCCTTGGAGCCATC

**AQP1-Eq14 R**

GATGTCGGTAACCGGTGTAACGGCACGTAAAGCAATTGCGAATCCTATCGCAACG

**AGLU1-piB6 F**

GCTGGTCGCGGCTACTGCTAGCTTCGCATTCTGTCCATCCAACCCTGGATTGCC

**AGLU1-piB6 R**

AGCGGGCTTGGCTAAAGTTAACTCGACGGAAATGGCGAGACCAAGAATTGCTCTCG

**AGLU1-Eq14 F**

CTACGTCACTCTGGCTATGACGTAGTTGATCTGTCCATCCAACCCTGGATTGCC

**AGLU1-Eq14 R**

GATGTCGGTAACCGGTGTAACGGCACGTAAAATGGCGAGACCAAGAATTGCTCTCG

**AGLU1_dsRNA F**

CTGTCCATCCAACCCTGGATTGCC

**AGLU1_dsRNA R**

AATGGCGAGACCAAGAATTGCTCTCG

**Aqp1_dsRNA F**

TCGCACAATGCCTTGGAGCCATC

**Aqp1_dsRNA R**

AGCAATTGCGAATCCTATCGCAACG

Supplementary Figures

**Figure S1**. **Characterization of the 50 most highly expressed small read biases and long read biased loci.**

**A)** 50 most high expressing long read (25-30nt) biased-loci. Heatmap shows distribution of reads between 16nt and 40nt. **B)** 50 most high expressing short read (19-23nt). si = siRNA sized, pi = piRNA sized. biased-loci Heatmap shows distribution of reads between 16nt and 40nt Yellow shows density of all reads. **C)** 3D scatterplot for the loci in A&B assessed by locus length, expression, and 1U bias. **D)** 50 top long (25-31nt) read loci where orange shows multi-mapping long reads, and violet uniquely-mapping long reads. **E)** 50 top short (19-23nt) read loci where orange shows multi-mapping short reads, and violet uniquely-mapping short reads.

**Figure S2.** **Visualization of RNA structure and small RNA expression at curated hpRNA loci.**

Top part of each panel depicts RNA structure with lines connecting one or more bases indicating pairing. Bottom panel is a density plot showing relative read depth across the locus. Red color indicates accumulation of 20-23nt reads that map to more than one position in the genome. Blue indicates 20-23nt reads that map uniquely to the locus. Yellow shows density of all read sizes.

**Figure S3**. **Proposed mechanism for piRNA/siRNA trigger.** A) Configuration of piRNA trigger where piRNA locus regions are modified to house sequence of the target gene. B) Single-stranded and double-stranded RNA piRNA triggers developed in the work

**Supplementary Datasets:**

**S1 Table (separate data file):** Annotated small RNA loci in this study (miRNA, cisNTAs, hpRNA, etc.)

**S2 Table (separate data file):** All small RNA loci identified in this study (total 3873 loci)

**S3 Table (separate data file):** Differential expression of the mRNA and small RNA loci (dsRNA fed vs control)
